# Characterization of envelope function of transmitted viruses circulating in Mbeya, Tanzania, and its impact on disease progression

**DOI:** 10.1186/1742-4690-9-S2-P144

**Published:** 2012-09-13

**Authors:** A Nofemela, G Bandawe, R Thebus, J Marais, L Maboko, M Hoelscher, C Williamson, Z Woodman

**Affiliations:** 1 University of Cape Town, Cape Town, South Africa; 2 Mbeya Medical Research Programme, Ministry of Health, Mbeya, Tanzania, United Republic of; 3 Department of Infectious Diseases and Tropical Medicine, Ludwig-Max, Munich, Germany; 4 Department of Molecular and Cell Biology, University of Cape Town, South Africa

## Background

An understanding of the biological characteristics of transmitted viruses provides important insights into HIV pathogenesis and informs vaccine development. The aim of the study was to characterize env function of transmitted viruses and its role in disease progression.

## Methods

Ten sequences were generated from single genome amplicons from 10 individuals at acute infection (range = 3 - 6 months post-infection) and the sequence representative of the consensus was cloned and functional env clones from subtypes C (n= 6), D (n =1) and recombinants CD (n = 2), AC (n = 1) were generated. Pseudovirions were generated, and entry efficiency in TZM-bl cells, tropism, dependency on CD4 and CCR5 using HEK 293 dual-inducible Affinofile cells, and sensitivity to entry inhibitors, was measured.

## Results

Half the envelope clones showed high levels of entry (52 -164% infection relative to Du151a, a reference env clone), and the remaining five had low entry efficiency (1- 18 %). We found an association between entry efficiency and viral load at 3 months (p = 0.0022) and 12 months postinfection (p = 0.0347). There was no significant correlation between entry efficiency and the IC50 of sCD4 (p = 0.5074), TAK779 (p = 0.4366) and enfurvitide (p= 0.5821), suggesting that the difference in entry efficiency was not due to CD4 and CCR5 binding, or membrane fusion. However, only 3/10 transmitted viruses from the group with high entry efficiency were able to infect cells with low of levels of CD4 and high levels of CCR5 receptors.

## Conclusion

Transmitted viruses have a range of entry efficiency in TZM-bl cells (with high CD4 and CCR5 levels) with high entry efficiency associated with higher viral loads. When the expression of CD4 was lowered, only three viruses were able to enter target cells, suggesting that transmitted viruses most likely target cells with high CD4 levels.

